# Activity competence among infants and toddlers with developmental disabilities: Rasch analysis of the Infant Toddler Activity Card Sort (ITACS)

**DOI:** 10.1186/s41687-021-00287-0

**Published:** 2021-01-21

**Authors:** Catherine R. Hoyt, Allison J. L’Hotta, Anna H. Bauer, Chih-Hung Chang, Taniya E. Varughese, Regina A. Abel, Allison A. King

**Affiliations:** 1 Program in Occupational Therapy, Washington University School of Medicine, 4444 Forest Park Blvd, MSC 8505-66-1, MO 63108 St. Louis, USA; 2 Institute of Informatics, Washington University School of Medicine, St. Louis, MO USA; 3 Department of Medicine, Washington University School of Medicine, St. Louis, MO USA; 4 Department of Pediatrics, Washington University School of Medicine, St. Louis, MO USA; 5 Department of Surgery, Washington University School of Medicine, St. Louis, MO USA; 6 Institute of Public Health, Washington University School of Medicine, St. Louis, MO USA

**Keywords:** Developmental delay, Caregiver reported outcome, Activity, Participation, Competence, Rasch analysis

## Abstract

**Background:**

Development is rapid in the first years of life. Developmental delays appearing during this critical period have the potential to persist throughout the child’s life. Available standardized assessments for this age record a child’s ability to successfully complete discrete skills but fail to capture whether the child incorporates those skills into daily routines that are meaningful to the child and family. The Infant Toddler Activity Card Sort (ITACS) is a newly developed photograph-based early intervention tool to measure the participation-related concept of activity competence using caregiver report. The purpose of the present study was to use Rasch analysis to determine if ITACS items comprehensively measure the construct of child activity competence.

**Results:**

A total of 60 child/caregiver dyads participated. The dichotomous caregiver-reported responses (present vs. absent) on the 40 individual ITACS items were used in Rasch analysis, and three iterations of the model were completed. The final model included 51 child/caregiver dyads and 67 ITACS assessments with a good spread of individual ability measure (6.47 logits). All items demonstrated adequate infit except for “sleeping” (range 0.68–1.54). Five items (sleeping, eating at restaurants, brushing teeth, crawling, and interact with pets) demonstrated high Mean Square (MNSQ) outfit statistics and one (take a bath) demonstrated low MNSQ outfit. ITACS items demonstrated a good spread of item difficulty measures (6.27 logits), and a clear ceiling was observed. Three activity items (smiling, breastfeeding, and playing with adults) were rarely endorsed as concerns. The activities most likely to be reported as challenging were “crying/communicating” and “going to school”. Person and item reliability statistics were adequate (0.79 and 0.80, respectively). The separation between individuals and between items were adequate to good (1.96 and 1.99, respectively).

**Conclusions:**

Findings indicate that ITACS items are measuring a unidimensional construct--activity competence in early childhood. The Rasch analysis of caregiver responses suggest that some activities are more likely to be considered challenging and may be important targets for intervention. These results provide evidence to further validate the ITACS as a caregiver report measure and support its use in the early intervention setting to facilitate caregiver driven goal development.

## Background

Developmental delay (DD) affects approximately one in seven children in the United States and presents a common challenge for caregivers and clinicians worldwide [1, 2]. Compared to their typically developing peers, children with disabilities require increased healthcare utilization and caregiver support with activities of daily living [3, 4]. Evaluating the impact of DD on children and their families is a primary concern for pediatric providers. Current DD measurement tools emphasize a child’s capacity to complete specific, age-appropriate skills associated with developmental milestones; however, these tools fail to incorporate caregiver input regarding the daily activities and routines that are most challenging for their children [5]. While developmental milestones are an important indicator of future development, healthcare providers must also assess a child’s participation and competence in activities and routines throughout the day to gain a comprehensive picture of the child’s everyday participation level [6]. Participation refers to a child’s purposeful involvement in everyday activities and routines, while competence refers to proficiency in a specific activity or task [7, 8]. Asking for caregiver perceptions of a child’s activity participation and competence can provide valuable information about child and family functioning. Additionally, caregiver involvement in the evaluation process can positively impact family functioning and the child’s developmental trajectory [9].

Providers are increasingly using tools that emphasize patient reported outcomes (PRO) to involve individuals in their own health management and to better understand meaningful effects of intervention [10]. While PRO reflect a single perpective from an individual, we can use results from PRO measures to compare commonalities across populations or diagnoses. Screening for DD is a top priority in healthcare for infants and toddlers, and concerns reported by caregivers can provide valuable insight into the daily functioning of children [11, 12]. Several tools exist to describe development (e.g. Ages and Stages Questionnaire) among infants and toddlers, however few describe functional abilities [13, 14]. The Routines Based Interview can guide conversations with caregivers about their child’s functioning, but the structure of the assessment does not provide a method for systematic scoring [15]. The Infant Toddler Activity Card Sort (ITACS) is a 40-item photograph based caregiver report tool of infant/toddler activity competence in which caregivers identify the activities that present challenges for their child [16]. Using a caregiver-report tool that uses photograph images rather than text alone, such as the ITACS, promotes evidence-based rehabilitation services focused on family-centered goal development and encourages a coaching approach to early intervention [17]. The ITACS was developed using conventional content analysis from caregiver interviews to determine the most common activities in which infants and toddlers regularly participate. Content validity was established by content experts [16]. While the primary purpose of the ITACS is to assess a child’s satisfactory participation and competence in family routines, it is also important to understand which activities most concern caregivers so that practitioners can focus on these concerns during intervention planning. The raw score obtained from the ITACS describes the number of activity challenges that a child experiences in the context of their family routines. A higher raw score indicates more difficulty participating in everyday activities. However, the participation-related concept of activity competence is a complex construct that we cannot directly measure. Since the ITACS uses caregiver report as a proxy measure of the child’s activity participation and competence, it is possible that a summed raw score may not accurately represent the child’s true abilities [18]. Understanding the perceived difficulty of individual ITACS items would provide essential information for its use in early intervention, allowing clinical providers to present items in an intentional order and to describe the child’s level of participation and competence deficit as mild or severe.

Item Response Theory (IRT) is a mathematical model that can evaluate how well a measurement tool captures latent traits, like participation and competence [19, 20]. IRT compares an individual’s response on a test item to the total responses for that item and uses goodness of fit to determine how well a construct measures a latent trait. Rasch measurement model, a one-parameter logistic IRT model, dictates that performance on a test item depends solely on the person’s ability and the item’s difficulty. Responses to these dichotomously scored (yes/no; present/absent; correct/wrong; true/false) items are converted into linear variables, thus Rasch Analysis (RA) utilizes a one-parameter logistic model to estimate the odds of an individual endorsing a dichotomously scored item [21, 22]. With RA, the level of difficulty of each item is determined and can be used to determine the order in which they are presented to the individual (from easiest to hardest). The RA techniques have been used to explain the meaning of survey scores, provide additional context to results, and improve the precision of measurement tools [23]. In this way, RA can ensure that a given assessment is the most accurate measure of a construct, as well as the most precise measure of change to that construct [23]. The purpose of the present study was to utilize RA to determine if the ITACS activity items represent varying levels of difficulty and to identify the most challenging daily activities of infants/toddlers within the ITACS measure, supporting further validation and refinement of this new pediatric assessment.

## Methods

A prospective design was used to gather responses from primary caregivers of children 0–3 years of age on the ITACS. Caregivers were asked to complete the ITACS twice, at baseline and 2 weeks later as a repeat assessment for a larger study. We anticipated that children with DD may have slight gains in ITACS raw scores if caregivers implemented any changes as a consequence of completing the ITACS. All study procedures were approved by the Institutional Review Board at Washington University School of Medicine.

### Participants

Primary caregivers (either the parent or individual who provided 8 h or more of childcare per day) of children 0–3 years of age with a DD were recruited to complete the ITACS. Following informed consent, caregivers provided demographic information and completed the ITACS using a tablet computer and Research Electronic Data Capture (REDCap), a secure, web-based application designed to support data capture for research studies [24]. The ITACS was programmed into REDCap to allow for flexible and efficient administration in community settings. Community based recruitment approaches and referrals were used to support a more diverse and representative participant cohort [25, 26]. This approach recruited children from multiple communities surrounding a mid-size city in the Midwest, improving external validity and generalizability.

### Measures

#### Demographic information

Caregivers provided information on demographics and if their child had a DD. Children were classified as having a DD if the caregiver reported any diagnoses associated with DD or indicated therapeutic service utilization, such as speech language pathology, occupational, or physical therapy.

#### Infant Toddler Activity Card Sort (ITACS)

Caregivers were presented with the ITACS items, which consisted of 40 photographs that corresponded with the most common daily activities of infants and toddlers (e.g. playing outside, eating finger food) [16]. The 40 items were derived from a qualitative analysis of caregiver reported activities that were then validated by experts using the Delphi method. A detailed description about the development of this measure has been previously published [16]. Items with their respective photos were administered in no particular order. Caregivers looked through the 40 ITACS activity items and were asked “Do you have concerns for your child related to (activity) for each item. Caregivers endorsed the items which presented challenges or concerns for their child in the context of everyday family routines. Of the endorsed items, caregivers selected up to five priority items that were most challenging for their children. For clinical purposes, caregivers were then asked to rank priority activities and specify their level of satisfaction (from “very dissatisfied” to “very satisfied”) in the child’s current level of functional participation and activity competence. Caregivers used the same Likert scale to specify their level of confidence in supporting the child in the priority activity. For the purpose of this study, only the dichotomous responses to the question “Do you have concerns for your child related to (activity)” for each of the 40 ITACS item were used in the RA. Thus, higher individual scores indicated more activity concerns for the child. Activity items that were endorsed more frequently were considered to be more challenging.

### Analysis

Demographic information was summarized using descriptive statistics. Dichotomous item responses were analyzed with RA using Winsteps version 4.4.3 [27]. By converting dichotomous responses to a linear scale, RA is able to quantify personal ability (i.e., the ability to perform a specific task) and item difficulty (i.e., the difficulty level of a specific task). Child participants with “greater” ability are less likely to have caregiver respondents endorse “easier” items as areas of concern. Caregivers endorsed concerns for approximately one out of every six items. Due to the large number of items for which caregivers did not endorse a concern, these data confounded the items rated as yes when RA was conducted with combined dichotomous and caregiver-reported performance ratings. When using the combined ratings, the RA models generated had large numbers of misfitting items and persons. A model with good person and item fit was not generated with the combined ratings despite multiple iterations of removing misfitting persons from the model. Therefore, only dichotomous responses were used to conduct the RA.

Three key components of Rasch modeling were the focus of these analyses: item and person fit, the Wright map with Rasch-derived measure scores, and reliability and separation statistics. To identify the optimal data-model fit, we conducted the following procedures. First, the variability of the model was determined with the mean square, such that a result of 1 indicates an accurate predictive model. This process is outlined below and was completed iteratively. The item difficulty was compared with the child participant’s ability using a logit (the sum of square item residuals) to determine the probability of an individual endorsing a particular item.

#### Item and person fit

In RA, fit statistics describe how accurately the person and item data fit the specified model by comparing the individual’s response to the model’s predicted response. Adequate mean-square (MNSQ) fit statistics for surveys fall between 0.6 and 1.4 and was used to assess which items demonstrated outfit [23]. Outfit statistics were used to identify outlier responses. When MNSQ values fell out of the recommended range, Z-standardized scores (ZSTD) were evaluated. A ZSTD higher than 2.0 requires further evaluation. When item outfit occurred, person outfit was examined and individuals with inconsistent (high outfit) responses were removed. This process was completed iteratively until item and person fit statistics fell into the acceptable MNSQ and ZSTD ranges or until no further statistical improvements were observed [23].

#### Wright map

The Wright map consists of two vertical histograms which provide a visual representation of each child participant’s ability alongside the difficulty of each item on the same linear scale [23, 28]. Less challenging items and children with lower ability (e.g. more activity concerns) are at the bottom of the map, while more challenging items are at the top of the map. An ideal Wright map would display an even distribution of the measures of persons and items. Gaps between the two item difficulty measures would indicate opportunities for additional item development. The child participants’ ages were correlated with their individual ability logit using a Spearman’s correlation using R version 3.5.3 [29].

#### Reliability and separation

Model reliability statistics indicate higher internally consistency when values are closer to 1.0. Person separation examines whether a test can differentiate between persons with high and low scores. Item separation examines the accuracy of item organization based on order of difficulty [18]. Separation statistics can range from 0 to infinity, with higher values indicating a greater degree of separation. A minimum separation of 1.5 is required for performing analyses at the individual level [23].

## Results

**Fig. 1:**
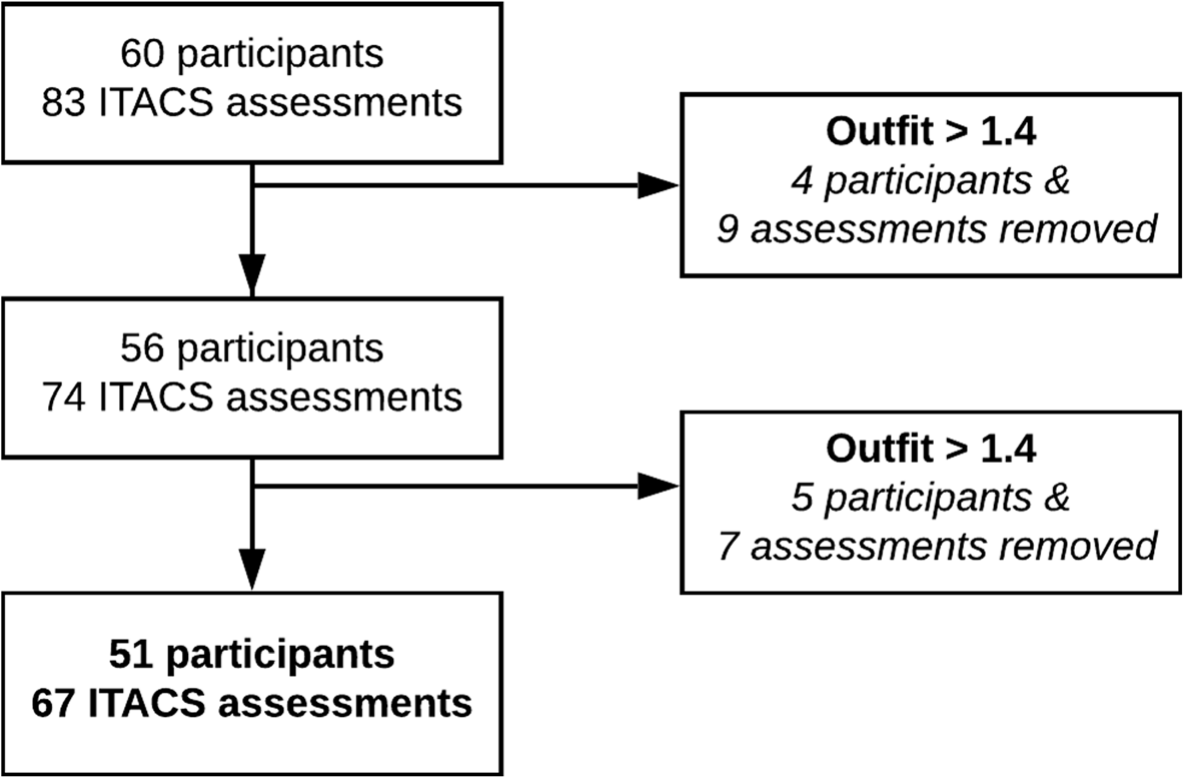
Recruitment and Model Fitting

A total of 60 caregivers of children with DD participated in this study and completed 83 ITACS assessments (23 completed both baseline and Time 2 ITACS). The number of participants and assessments included in each Rasch model are outlined in Fig. 1. ITACS assessments were removed based on outfit. The third Rasch model demonstrated sufficient fit to the data and consisted of responses from 51 caregiver/child dyads (Table 1). Nine participants were removed due to high person/model outfit statistics. Most caregivers self-reported that their child had a DD (88%). The remaining 6 (12%) had a diagnosis of sickle cell disease (SCD), which is acknowledged to be associated with DD at young ages and were thus included in this cohort [30, 31]. We were unable to confirm the diagnoses associated with DD for the remaining participants as a consequence of our community recruitment approach. Of these 51 dyads, 16 (31%) completed the ITACS a second time for a total of 67 assessments included in the final model. Based on previous reports, this sample size is adequate to have 99% confidence that the estimated item difficulty is within one unit of the true item measure score [32].

**Table 1:** Participant Demographics (*n* = 51)

	Caregiver ***n (%)***	Child ***n (%)***
Gender		
Female	45(88)	22(43)
Male	5(10)	29(57)
Not Reported	1(2)	0(0)
Other	0(0)	0(0)
Age (years or months)		
Mean (*range)*	33.02*(19–61) yr.*	22.76*(5–42) mo.*
Ethnicity		
Black/African American	26(51)	27(53)
White/Caucasian	21(41)	24(47)
Asian	3(6)	5(10)
American Indian/Alaskan Indian	1(2)	3(6)
Pacific Islander/Native Hawaiian	0(0)	0(0)
Other	0(0)	0(0)
Hispanic/Latinx	2(4)	3(6)
Caregiver Type		
Biological Mother	36(71)	–
Biological Father	5(10)	–
Grandparent	2(4)	–
Other	7(14)	–
Not Reported	1(2)	
Marital Status (*n* = 197)		
Married	28(55)	–
Single	21(41)	–
Divorced	1(2)	–
Not Reported	1(2)	
Highest Level of Education Completed		
< High School	2(4)	–
High School/GED	29(57)	–
College or Higher	20(39)	–
First Time Caregiver		
Yes	18(35)	–
Not Reported	5(10)	

### Fit statistics

Item infit MNSQ statistics ranged from 0.68 to 1.54 and outfit MNSQ ranged from 0.34 to 1.90 (Table 2). All items demonstrated adequate infit except for “sleeping”, which demonstrated borderline infit MNSQ and ZSTD. In the final model, five items (sleeping, eating at restaurants, brushing teeth, crawling, and interact with pets) demonstrated high MNSQ outfit and one (take a bath) demonstrated low MNSQ outfit. Further investigation of outfit ZSTD indicated the items “sleeping” and “eating at restaurants” performed unexpectedly for some participants as identified through ZSTD values outside the recommended range of − 2 to 2. These two items are considered somewhat more challenging, as indicated by their item measure score of 0.79. However, some participants whose scores indicate the child had lower ability (higher ITACS raw scores) did not identify these more difficult items as areas of concern for their child. Not endorsing concerns on these more challenging items is identified as an unexpected response, resulting in item outfit.

**Table 2:** ITACS Item Statistics

Item	Measure	Infit MNSQ	Infit ZSTD	Outfit MNSQ	Outfit ZSTD
Crying/communicating	2.11	0.93	−0.50	1.08	0.44
Going to school	1.52	0.87	−0.94	0.76	−1.18
Walking	1.52	1.05	0.41	0.92	−0.30
Using the potty	1.32	0.89	−0.70	0.91	−0.32
Running	1.12	1.11	0.76	1.00	0.08
Reading books	0.79	1.07	0.46	1.01	0.13
Get dressed	0.79	0.83	−1.04	0.69	−1.09
Sleeping	0.79	1.54	2.91	1.90	2.51
Eating at restaurants	0.79	1.25	1.46	1.84	2.39
Play with puzzles	0.68	1.03	0.22	0.84	−0.42
Interact with pets	0.56	1.28	1.54	1.44	1.27
Social interactions	0.44	1.14	0.81	1.30	0.87
Crawling	0.44	1.17	0.95	1.53	1.38
Coloring/drawing	0.31	0.92	−0.38	0.98	0.07
Climbing on play equipment	0.31	1.02	0.19	1.15	0.49
Spoon feeding	0.31	1.00	0.08	0.75	−0.58
Pretend play	0.18	0.89	−0.50	0.73	−0.57
Play with tablet	0.18	0.76	−1.24	0.52	−1.25
Bottle feeding	0.18	0.96	−0.13	1.03	0.22
Finger feeding	0.18	1.17	0.86	1.15	0.48
Brushing teeth	0.18	1.19	0.95	1.66	1.46
Playing	0.04	0.77	−1.14	0.55	−1.06
Using a cup	−0.11	0.92	−0.31	0.71	−0.49
Tummy time	−0.27	1.16	0.71	1.40	0.84
Running errands with you	−0.27	0.80	−0.80	0.46	−1.10
Diaper changing	−0.44	1.02	0.16	1.13	0.41
Playing outside	−0.44	1.13	0.56	0.77	−0.23
Attending religious services	−0.63	0.94	−0.11	0.58	−0.55
Help with cooking/meals	−0.63	0.87	−0.37	0.91	0.07
Play with blocks	−0.84	0.85	−0.39	0.38	−0.89
Ride in the car or use transportation	−0.84	0.85	−0.40	0.50	−0.61
Take a bath	−0.84	0.68	−1.06	0.34	−1.02
Watching TV	−1.08	1.16	0.55	0.77	−0.04
Play with siblings	−1.08	0.92	−0.13	0.52	−0.44
Music/singing	−1.70	1.03	0.22	0.80	0.19
Going for walks	−1.70	0.93	0.02	0.37	−0.37
Swinging	−1.70	0.80	−0.28	0.41	−0.29
Playing with adults	−2.17	0.89	0.01	0.54	−0.01
Breastfeeding	−4.16	Minimum Measure			
Smiling	−4.16	Minimum Measure			

### Item difficulty

**Fig. 2:**
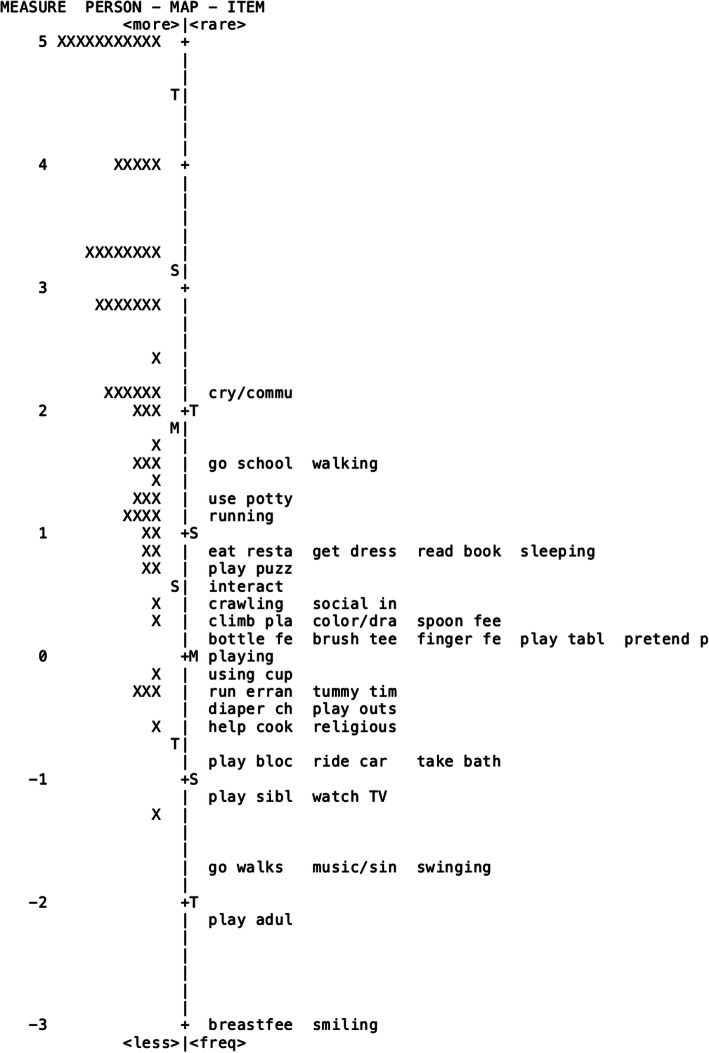
Wright Map of Participants and Items

Estimates of item difficulty are represented by the Rasch-derived measures presented in Table 2. Higher measure values indicate more difficult items or items more frequently endorsed as areas of concern while lower measure values represent easier/less frequently endorsed items. The hierarchical organization of item difficulty is also represented in the Wright Map (Fig. 2).

The ITACS items vary in difficulty. Some items were rarely endorsed as concerns (e.g. smiling, breastfeeding, playing with adults), while other items were frequently endorsed as challenging (e.g. going to school). Items related to feeding (e.g. finger feeding) and play (e.g. play with tablet) clustered around the mean item measure. These feeding and play items had similar difficulty to the overall measure and cluster around the mean (+M on the Wright map).

### Wright map

The Wright map (Fig. 2) demonstrated a good spread of items (6.27 logits) and persons (6.47 logits), but also demonstrated a clear ceiling effect. Approximately half of the children rated by their caregivers were mapped at a higher ability level than the most difficult item (crying/communicating; an indicator of the child’s ability to communicate their wants and needs). The observed ceiling effect indicates the ITACS may not have items to capture the ability level of more able infants and toddlers. Eleven (16%) participants did not endorse any items as presenting challenges for their child. These participants are represented on the top left of the map. The most challenging activities identified were “communicating wants/needs”, “walking”, and “independent community outings” (e.g. going to daycare/school). No participants endorsed “breastfeeding” or “smiling” as concerns.

The Wright map was inspected for patterns in participant ability level based on age. Age was not a significant predictor of ability on the ITACS (S = 49,453, *p* = 0.80). This assessment was developed to evaluate participation and activity competence of infants and toddlers, thus response patterns based on age were not expected.

### Reliability and separation

Person and item reliability and separation statistics improved with each model iteration. Person (0.79) and item (0.80) reliability statistics were adequate (Table 3). The separation between individuals and between items was classified as adequate to good (1.96 and 1.99, respectively). Good person separation indicates the ITACS differentiates between infants and toddlers of different ability levels. Good item separation suggests a hierarchy of difficulty from easier to harder items and suggests that ITACS activity items cover a range of skills [18].

**Table 3:** Reliability and Separation of ITACS Items

	Separation	Reliability
*Person*	1.96	0.79
*Item*	1.99	0.80

## Discussion

In this present study we collected data from 60 caregivers of children with DD and used RA to further validate the ITACS as an effective early intervention tool. The results of the RA allow us to hierarchically describe caregiver reported infant/toddler participation and competence in everyday activities and understand which activities are most challenging for this age group. We report good item fit and strong separation between person and item responses. By converting ordinal responses to an interval scale, RA describes the difficulty of each of the 40 ITACS items in relation to the overall assessment tool. The analysis revealed that the items represent a good distribution of difficulty. Future studies may incorporate this information when considering the presentation order or determining if additional items are needed. These findings provide valuable information about the ITACS and its practical use as an early intervention tool for pediatric providers.

Since the ITACS is designed to measure the participation-related construct of infant/toddler competence to complete everyday routines, we expected items to fit closely together. Based on the results presented in the Wright map, the most frequently endorsed item was “crying/communicating”, which we attribute to caregivers placing a high value on meeting their child’s needs. Many caregivers endorsed “walking” as an area of concern, which we attribute to the high priority many caregivers place on this developmental milestone. We were surprised to find that many of the activities identified as the most difficult may require complex social interaction, such as “going to school” or “eating at restaurants”. Picture-based tools such as the ITACS may aid in identifying early indicators of activity participation challenges resulting from social interaction. We also found that some activities that are often considered to be more challenging (e.g. helping to cook, attending religious activities) had low item measure scores. As a consequence of PRO, these activities may have had artificially low item measure scores because caregivers had not attempted these activities with children who exhibited lower ability. The impact of disability on activity competence varies widely between diagnoses and can be heavily influenced by the home environment [33]. The ITACS covers a broad range of skills and the frequency of reported challenges was likely influenced by the exposure the child had to a specific activity in their daily environment. We observed that many of the items related to feeding and play clustered together around the mean. We identified that concordant items had similar difficulty, further validating the ITACS tool. The items “smiling” and “breastfeeding” were never endorsed as a concern by caregivers. The activity of “breastfeeding” may not have been endorsed because children were bottle fed or had already transitioned to table food. It is not surprising the activity of “smiling” was not identified as a concern, as it is a skill that typically develops in the first months of life, and our cohort ranged in age from 5 to 42 months. Examining the Wright map and fit statistics allows for an examination of the overall measures as well as each item. Insufficient fit (< 1.4) and potential item redundancy (same Rasch measure score) suggest that some items be removed from the ITACS. However, since we intentionally developed the ITACS items with caregiver input and expert review to cover the most common activities of infants/toddlers [16], we determined that removing items would weaken the integrity of the tool.

As with any study, there are limitations to this analysis. The ITACS scores are based on caregiver report of their child’s daily activity participation and competence. It is possible that caregivers either under or over reported concerns [34]. The ITACS reports a total of three scales, but for this study we analyzed the scale that provides dichotomous responses indicating whether or not an activity item presents a concern to the caregiver. While this scale best fit the parameters of a RA, future studies may consider incorporating the caregiver reported performance and confidence scales. We observed a clear ceiling effect of the ITACS, indicating that some caregivers reported no activities as a concern. We believe that this ceiling effect reflects children who are not experiencing substantial DD and may correlate to higher scores on traditional developmental measures. Additional studies are needed to determine the relationship of the ITACS to standardized developmental measures. We noticed that the majority (66%) of caregivers of children with SCD reported no activity concerns. This finding may reflect the fact that these caregivers did not self-report that their child had a DD.

Future studies should consider if the ITACS items have differential item functioning, indicating that item responses would vary between different populations. More work is needed to describe the concurrent validity of the ITACS and determine the implementation approach for use in early intervention. These results will allow for future studies to consider opportunities for additional items to be included to improve the range of difficulty across items. Future item development should be conducted in collaboration with both caregivers and experts for validation of the activity items and corresponding images.

## Conclusion

In conclusion, we report the results from a RA of caregiver reported activity competence concerns among infants and toddlers with DD using the ITACS. These findings provide insight into what daily activities are perceived by caregivers to be the the most challenging for their children and could be primary targets for intervention services. The ITACS items demonstrated good fit and separation, providing additional evidence to support the use of this caregiver report measure in the early intervention setting. As early intervention adopts caregiver coaching as a model for family-centered services [35], it is essential that measurement tools are developed to incorporate caregiver goals and perceptions of intervention outcomes.

## Data Availability

The datasets used during the current study are available from the corresponding author on reasonable request.

## Abbreviations

DD: Developmental delay
PRO: Patient reported outcomes
IRT: Item Response Theory
ITACS: Infant Toddler Activity Card Sort
RA: Rasch analysis
SCD: Sickle Cell Disease
